# Editorial: Applying large animals for developmental study and disease modeling

**DOI:** 10.3389/fcell.2023.1225060

**Published:** 2023-05-30

**Authors:** Lin Yuan, Feng Yue, Jacek Z. Kubiak, Sen Wu, Yongye Huang

**Affiliations:** ^1^ Laboratory of Research in Parkinson’s Disease and Related Disorders, Health Sciences Institute, China Medical University, Shenyang, China; ^2^ Key Laboratory of Biomedical Engineering of Hainan Province, School of Biomedical Engineering, Hainan University, Haikou, China; ^3^ Faculty of Medicine, Dynamics and Mechanics of Epithelia Group, CNRS, Institute of Genetics and Development of Rennes (IGDR), University Rennes, Rennes, France; ^4^ Laboratory of Molecular Oncology and Innovative Therapies, Military Institute of Medicine (WIM-PIB), Warsaw, Poland; ^5^ State Key Laboratory of Animal Biotech Breeding, College of Biological Sciences, China Agricultural University, Beijing, China; ^6^ Sanya Institute of China Agricultural University, Sanya, China; ^7^ Key Laboratory of Bioresource Research and Development of Liaoning Province, College of Life and Health Sciences, Northeastern University, Shenyang, China

**Keywords:** gene editing, CRISPR/Cas9, embryo, development, pig, rabbit, monkey

## Introduction

**FIGURE 1:**
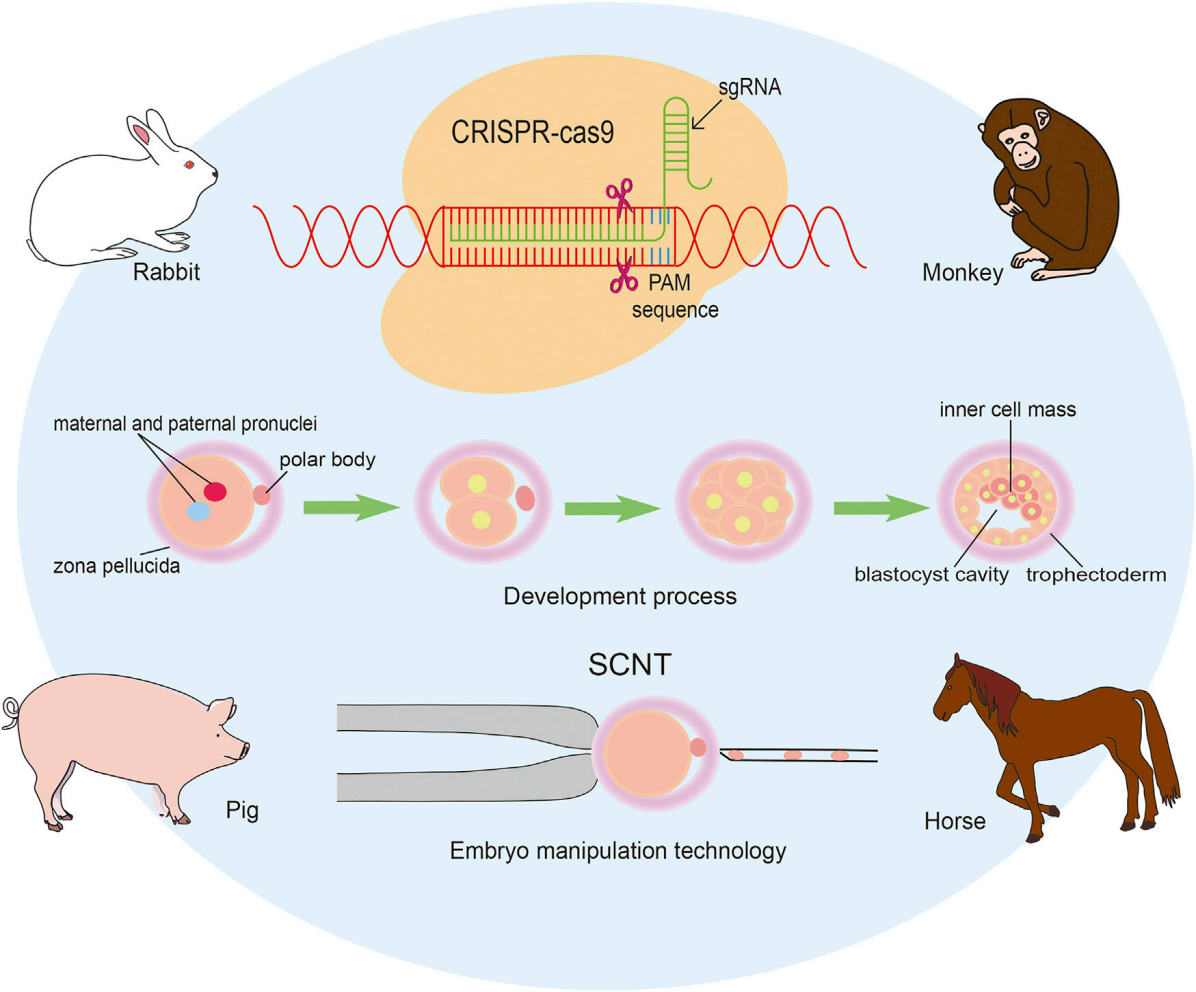
Applying genomic editing technology and somatic cell nuclear transfer to generate large animal models.

We have a great pleasure to organize the Research Topic “Applying large Animals for Developmental Study and Disease Modeling”. It is well known that large animals show similarities in many physical and pathological characteristics with human beings and have numerous advantages in developmental and medical research. In recent years, an important breakthrough has been achieved in gene editing technique, including ZFN, TALEN, CRISPR/Cas9, and so on. Especially for the emergence of CRISPR/Cas9 mediated gene modification strategies, various types of human diseases have been recapitulated in large animal models. It has to be mentioned that the progress in establishing embryonic stem cells and inducible pluripotent stem cells is also critical for the application in diseases modeling and therapies. In our Research Topic more than ten papers have been collected, and most of them focus on pig, monkey, rabbit and horse (Figure 1).

## Pig

The pig, as one of the most common livestock, exhibits several advantages in biomedical research. These are: comparable human and pig body sizes, similar anatomical and physiological characteristics, genomic composition and ordinary diets (Hou et al., 2022). It makes the pig a promising alternative animal model for humans. Rapid growth rate, early sexual maturity, short generation intervals, high number of offspring per litter, and standardized breeding techniques, also make easier the application of pigs in the study on human diseases. In recent decades, the advances in technologies contribute to the development of genetically engineered pig models of human diseases. The emergence of somatic cell nuclear transfer (SCNT) and reliable genome editing techniques make possible for the generation of porcine models of human diseases. The efficacy of porcine SCNT has been largely improved and several kinds of small molecular inhibitors have been applied to enhance the developmental competence of SCNT embryos (Ouyang et al., 2021; Hou et al., 2022). CRISPR/Cas9-mediated gene editing strategies are widely used in the establishment of porcine models of human diseases. Various genomic genetically engineered pigs have been generated, including chimeric gene knock in, point mutations, large genome fragment deletions, multifunctional live cell sensors, *etc*. One of the most promising potential application of pigs in the biomedical field is xenotransplantation. In 7 January 2022, the medical school of the University of Maryland in the United States conducted the first-ever life-saving cardiac xenotransplantation and it was successful in extending the patient’s life for about 8 weeks (Rothblatt, 2022). The breakthrough sheds light on xenotransplantation using porcine organs as donors. Actually, corneas, lungs, nerve cells, kidneys, livers, and islets of pigs are also potential candidates for xenotransplantation. In the Research Topic, Junliang Li et al. evaluated the methylation status of IG-DMR and gene expression profile in the DLK1-DIO3 region (Li et al.); Lin et al. revealed that intravenous injection of AAV9-GFP could result in widespread expression of transgene in various porcine organs (Lin et al.); Hilansi Rawat et al. found that porcine expanded pluripotent stem cells could differentiate into cardiovascular progenitor cells, functional cardiomyocytes, epicardial cells and epicardial-derived cells, and they established an enhanced system for whole-embryo culture allowing *ex utero* development of porcine post-implantation embryos from ED14 up to ED17 (Hilansi et al.); and many papers review the progress of applying pigs in xenotransplantation and biomedical research.

## Nonhuman primates

Nonhuman primates (NHP) emerge increasingly as excellent models for translational research, due to even closer proximity to human beings in terms of physiology, biochemistry, immunology, pathology and genetic evolution. For example, NHP models are widely used in studies on Severe Acute Respiratory Syndrome Coronavirus 2 (SARS-CoV-2). In fact, NHP models of HIV, ZIKV or Ebola virus infection mirror closely the pathogenesis in human patients. SCNT monkeys have been successfully produced using fetal fibroblasts as donor cells in recent years (Liu et al., 2018). Furthermore, BMAL1 gene-edited monkeys via the CRISPR/Cas9 technique were generated with both intracytoplasmic sperm injection and SCNT method (Liu et al., 2019; Qiu et al., 2019). Cynomolgus monkey blastoids resembling blastocysts in morphology and transcriptomics using naive ESC develop to embryonic disk with the structures of yolk sac, amnion cavity, chorionic cavity and primitive streak via prolonged *in vitro* culture (Li et al., 2023), making it possible to investigate primate embryonic development without the same ethical concerns associated humans. Various NHP models of human diseases have been established in recent years, including bilaterally delivering synthetic Aβ oligomers into the cerebral parenchyma of cynomolgus monkeys to drive early pathological progression of Alzheimer’s disease (Yue et al., 2021). However, in the near future, researchers might face monkey shortage crisis (Grimm, 2023). In the Research Topic, Liang et al. summarizes the recent progress in using genomic editing technology in the establishment of NHP models and discusses the factors limiting the wide application of NHP models of human diseases (Liang et al.).

## Rabbit

Rabbit is another commonly used experimental model for investigating equivalents of human diseases. With the emergence of microinjecting, SCNT and genomic editing technology, a large number of rabbit models of human diseases were established. The pronuclear microinjection is still the most common method in the generation of transgenic rabbit models. Novel genomic editing technologies, such as CRISPR/Cas9, remarkably promote precision in rabbit genome manipulation (Song et al., 2020). In previous studies, CRISPR/Cas9 technique was applied to introduce mutation of αA-Crystallin or GJA8 gene to induce congenital cataracts in Rabbits (Yuan et al., 2016; Yuan et al., 2017). Recently, SpRY-ABEmax mediated base substitution has been used to generate YIPF5 (p.W218R) mutation to generate rabbit primary microcephaly model, which precisely recapitulate the typical symptoms of human primary microcephaly (Liu et al., 2023). With recent technological innovations in genomic editing techniques, rabbit models will certainly play a much more important role in the study on human diseases. In our Research Topic, a carrageenan-induced abdominal aortic adventitial inflammatory model in hypercholesterolemic rabbits is described (Chen et al.). It determines the role of MMP-12 secreted from adventitial macrophages in the pathogenesis of this diseases (Chen et al.).

## Horse

Horses are commonly used as animal models for studying several human diseases, including neurodegenerative diseases, mental and behavioral disorders, neuropsychiatric disorders and spontaneous sepsis. Using SCNT and genomic editing technology to generate horse models of human diseases seems to be less attractive than porcine and rabbit models. However, the rapid development in horse models have been achieved in recent years. A recent report has revealed that a total of 12 SCNT foals were born (Cortez et al., 2023), and gene-edited horse embryos have been generated by CRISPR/Cas9 technology (Maniego et al., 2022). In our Research Topic, Neil Marr et al. utilized immunolabelling for CD146 to determine horse tendon cell population, providing the intrinsic evidences for the relationships between local interfascicular matrix vascular and basement membrane constituents (Marr et al.).

In conclusion, the Research Topic “Applying large Animals for Developmental Study and Disease Modeling” delivers a whole plethora of excellent examples of the fascinating progress made recently in this field of biomedical research.
